# Novel Synthesis of Zinc Oxide on Cotton Fabric by Cathodic Cage Plasma Deposition for Photocatalytic and Antibacterial Performance

**DOI:** 10.3390/ijms251810192

**Published:** 2024-09-23

**Authors:** Rayane Saory Medeiros dos Santos, Muhammad Naeem, Anderson Lucas da Silva, Michelle De Medeiros Aires, Rômulo R. Magalhães de Sousa, Thércio Henrique de Carvalho Costa, Hugo Alexandre Oliveira Rocha, Maria Celeste Nunes De Melo, Michelle Cequeira Feitor

**Affiliations:** 1 Textile Engineering Post-Graduation, Federal University of Rio Grande do Norte, Natal 59078-970, RN, Brazilmichellemaires@gmail.com (M.D.M.A.); michelle.feitor@ufrn.br (M.C.F.); 2 Department of Physics, Women University of Azad Jammu and Kashmir Bagh, Bagh 12500, Pakistan; 3 Textile Engineering Department, Federal University of Rio Grande do Norte, Natal 59078-970, RN, Brazil; 4 Mechanical Engineering Department, Federal University of Piauí, Teresina 64049-550, PI, Brazil; romulorms@gmail.com; 5 Mechanical Engineering Department, Federal University of Rio Grande do Norte, Natal 59078-970, RN, Brazil; thercio.costa@ufrn.br; 6 Department of Biochemistry, Federal University of Rio Grande do Norte, Natal 59078-970, RN, Brazil; hugo.rocha@ufrn.br; 7 Department of Microbiology and Parasitology, Federal University of Rio Grande do Norte, Natal 59078-970, RN, Brazil; celmelo@gmail.com

**Keywords:** zinc oxide nanoparticles, cotton fabric, antibacterial properties, photocatalytic properties, cathodic cage plasma deposition

## Abstract

Cotton fabrics with zinc oxide (ZnO) coating are of significant interest due to their excellent antibacterial performance. Thus, they are widely in demand in the textile industry due to their medical and hygienic properties. However, conventional techniques used to deposit ZnO on fabric require long processing times in deposition, complex and expensive equipment, and multiple steps for deposition, such as a separate process for nanoparticle synthesis and subsequent deposition on fabric. In this study, we proposed a new method for the deposition of ZnO on fabric, using cathodic cage plasma deposition (CCPD), which is commonly used for coating deposition on conductor materials and is not widely used for fabric due to the temperature sensitivity of the fabric. The effect of gas composition, including argon and a hydrogen–argon mixture, on the properties of ZnO deposition is investigated. The deposited samples are characterized by XRD, SEM, EDS, photocatalytic, and antibacterial performance against *Staphylococcus aureus* and *Pseudomonas aeruginosa* bacteria. It is observed that ZnO-deposited cotton fabric exhibits excellent photocatalytic degradation of methylene blue and antibacterial performance, specifically when a hydrogen–argon mixture is used in CCPD. The results demonstrate that CCPD can be used effectively for ZnO deposition on cotton fabric; this system is already used in industrial-scale applications and is thus expected to be of significant interest to garment manufacturers and hospitals.

## 1. Introduction

Cotton fabric exhibits unique properties, including durability, relatively low cost, good dyeability, hydrophilicity, and biodegradability, and is widely used in numerous applications [1,2]. However, it still shows some intrinsic drawbacks, like microbial attacks on the cotton fabric due to fibrous structure, induction of crease, and loss of mechanical strength during the finishing process [3]. Modern cotton fabric should have certain features, including being hygienic, durable, functional, smart-looking, and comfortable [4]. Achieving these exceptional properties is challenging, and nanotechnology is extensively applied for surface modification of cotton fabric [5]. In this process, numerous nanoparticles are included in the finishing stage of textile material, which, as a result, resolves the above-described issues and also modifies the functional properties [6,7]. In addition, the nanomaterial-coated functional textiles can prevent the detrimental effects of ultraviolet (UV) rays, which, as a result, prevent skin cancer and other diseases associated with these radiations [8]. 

Among the various metal and metal oxide nanoparticles, zinc oxide (ZnO) stands out for its unique properties, including antimicrobial and antifungal activities [9]. ZnO is a versatile nanomaterial offering exclusive optical, antibacterial, electrical, self-cleaning, and UV-blocking properties [7]. Its high optical absorption in the ultraviolet regions (UVA 315–400 nm and UVB 280–315 nm) is particularly valuable for its antibacterial response [8]. Moreover, ZnO is FDA-approved as a biosafe and biocompatible material for medical applications, making it an effective antimicrobial agent [9]. Besides this, ZnO nanomaterials are advantageous over silver nanomaterials in several aspects, including their relatively low cost, stable nature, and white/colorless appearance [10,11]. 

Several authors have attempted to deposit ZnO nanoparticles on cotton fabric using numerous chemical and physical routes to prepare a low-cost nanotextile with self-cleaning and antibacterial properties. These routes include the pad–dry–cure method [12], layer-by-layer deposition [13], dip-coating method [14], wet-chemical route [15], photosonochemical technique [16], and chemical grafting method [17]. Hu et al. [18] fabricated ZnO cotton fabric using the surface micro-dissolution method and found improvement in antibacterial performance and radiation barrier properties. Patil et al. [10] reported that the deposition of ZnO on cotton fabric assisted with sonosynthesis in a single-step process. They found that it can improve the antimicrobial performance of fabric. Tania et al. [19] used a mechanical thermo-fixation technique for coating ZnO nanoparticles on cotton fabric. They obtained bacterial reduction, UV protection ability, and a positive role of binder on these properties. Javed et al. [20] deposited ZnO nanoparticles on cotton fabric by ultrasonically assisted in situ deposition, and the fabric showed excellent antibacterial properties, self-cleaning, and ultraviolet protection factor (UPF). Recently, Verbic et al. [21] used the green in situ method to synthesize ZnO nanoparticles and subsequently deposited them on cotton fabric, and the resultant fabric exhibited excellent UV protection. Unfortunately, most of these nanofabric preparation techniques involve multiple-step processing, which requires the synthesis of nanoparticles and their subsequent deposition on cotton fabric. The nanoparticles are typically synthesized by wet-chemical routes, which have various limitations. These include aggregation of nanoparticles, usage of toxic reducing agents, uncontrolled size and morphology of nanoparticles as it is dependent on reducing agents, unnecessary use of water for washing, environmental threats caused by toxic chemicals, and time consumption in drying of nanoparticles [22]. Therefore, environmentally friendly and consistent approaches are essential for the deposition of nanoparticles on cotton fabric, which should not involve the wet-chemical route of nanosynthesis and multiple-step processing.

The cathodic cage plasma deposition (CCPD) system was introduced around two decades ago, mainly to improve the surface properties of metallic materials, including steel [17]. It exhibits several advantages over conventional treatment techniques, including environmental suitability and relative cost-effectiveness. Its working mechanism [23,24,25] is based on the bombardment of energetic ions formed in the plasma on the surface of a metallic screen (termed a cathodic cage), the resultant sputtering, and redeposition of cage material on the surface of the sample to be treated. Several authors have used this technique in the literature to deposit numerous materials on metallic samples, such as niobium [26], copper [27], chromium [28], aluminum [29], Hastelloy [30], titanium [31], vanadium [32], and molybdenum [33] on a steel substrate. However, the use of this technique for the processing of nonmetals and insulators is limited in the literature [27]. 

In a previous report [27], we applied CCPD to deposit copper on the polytetrafluorethylene (PTFE) samples, assisted with a copper cathodic cage, and obtained improvement in surface properties. However, it is essential to mention that fabric samples are more sensitive to temperature, which is significant in CCPD and damages the fabric material. Formerly [34], we introduced the idea that fabric materials may be placed outside the cathodic cage and that copper oxide could be deposited on polyamide and polyester fabrics. In this work, keeping in mind the importance of ZnO nanomaterial-coated fabric and its possible use for numerous medical applications, it is deposited on cotton fabric by CCPD. The role of processing gas, including argon and hydrogen–argon, on the ZnO deposition and resultant photocatalytic and antibacterial performance is examined. 

## 2. Results and Discussion

**Figure 1:**
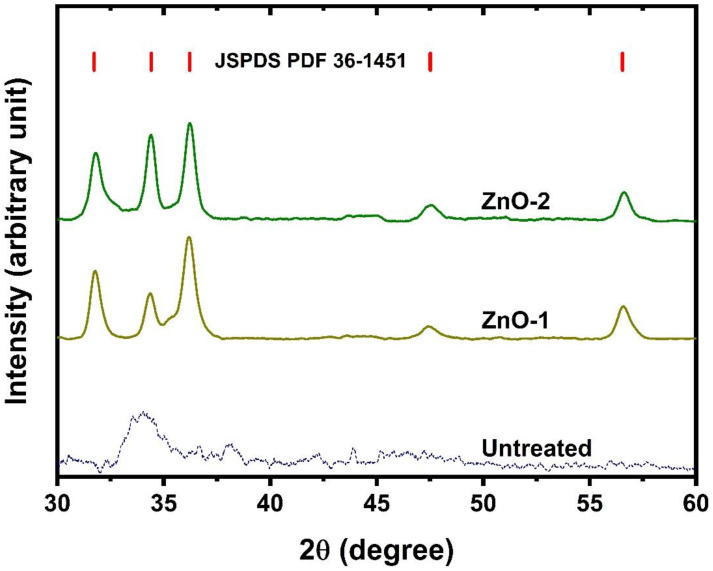
X-ray diffraction pattern of cotton fabric with and without ZnO deposition.

The cotton fabric is treated by cathodic cage plasma deposition (CCPD), using a steel cathodic cage with zinc oxide rings inserted in the cage’s holes to deposit ZnO on the sample’s surface. Figure 1 presents the X-ray diffraction pattern of untreated cotton fabric and samples with ZnO deposition at various gas compositions. It shows that fabric with ZnO deposition is crystalline, irrespective of gas composition, and peaks correspond to the face-centered cubic structure of ZnO (JCPDS-36-1451) [35,36]. The peaks appear at 2θ = 31.76°, 34.37°, 36.13°, 47.47°, and 56.41°, which correspond to various planes (100), (002), (101), (102), and (110) of the hexagonal wurtzite phase [37,38]. The XRD pattern of cotton fabric shows a peak around 2θ = 34.5°, which resembles the typical cellulose I (Iβ) peak, which follows the literature [39]. The results are well agreed with previous reports in which other techniques are applied to deposit ZnO on cotton fabric [10,40,41,42]. The XRD reveals the presence of ZnO nanoparticles on fabric samples by cathodic cage plasma deposition obtained by single-step deposition. In this system, separate steps are not required to synthesize nanoparticles and subsequent deposition on fabric; thus, it is favorable for preparing ZnO-coated fabric.

To assess the durability and long-term stability of ZnO coating on fabric, a wash test was performed following the AATCC 0061-2010 method of accelerated wash. According to the standard, each wash equals five times the domestic washes. After 60 wash cycles, the XRD pattern still reveals the presence of ZnO on the fabric (spectra not shown), which indicates good adhesion with the fabric.

**Figure 2:**
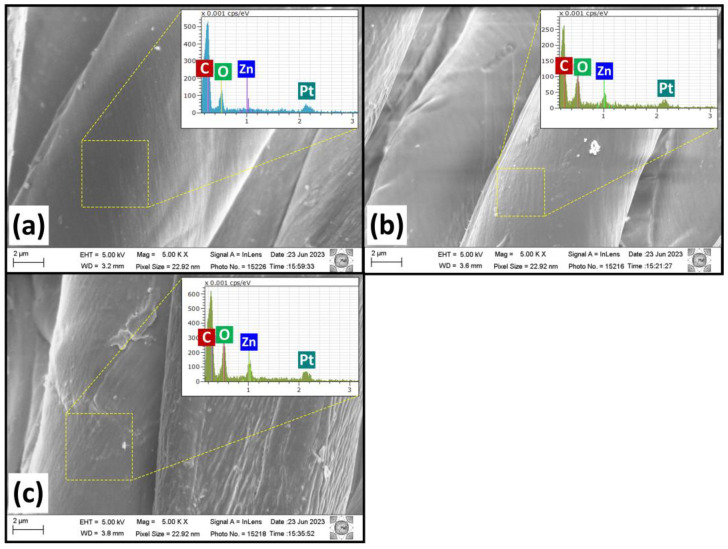
SEM images along with EDS of cotton fabric: (**a**) untreated material, (**b**) ZnO-1 sample, and (**c**) ZnO-2 sample. (The microscope magnification of 5 kX and scale bar of 2 μm).

Figure 2 presents the surface morphology of cotton fabric with and without ZnO deposition. The untreated sample (Figure 2a) shows a characteristic ridge and a clean and smooth surface appearance. On the other hand, when samples are treated (Figure 2b,c) by CCPD, these ridges on untreated samples vanish irrespective of gas composition, and a rough surface appears [41,43]. The SEM image clearly shows that the surface roughness of the untreated sample is changed by ZnO deposition. When the coating is deposited by adding hydrogen gas in argon (Figure 2c), particles are covered on the entire fabric surface. The change in deposition rate by changing the gas composition can be ascribed as follows: as proposed in several mechanisms of CCPD [24,25,44], the coating deposition in this system is based on multiple steps involving the sputtering of cage material due to the bombardment of energetic ions on Zn rings, and then redeposition on the samples’ surface. When hydrogen gas is added to argon plasma, as the hydrogen gas has a low ionization cross-section, the electron temperature increases [45]. As a result, it causes an increase in the number of plasma energetic ions impacting the surface of the cathodic cage, and thus, the sputtering rate increases [46,47]. The EDS spectra of untreated cotton fabric and ZnO-deposited fabric for elemental analysis are also presented in Figure 2. The cotton fabric with and without ZnO deposition shows the presence of carbon and oxygen elements, which can be ascribed to the existence of long-chain cellulosic units in the fabric [48,49]. In addition, EDS shows the occurrence of zinc (6.7 and 13.4%) and oxygen elements (40% and 35%) on cotton fabric treated by the CCPD technique. As no additional elements are detected (except platinum, which is coated for analysis of non-conductive samples to avoid the charging effect), the ZnO deposition by this technique is highly pure, as observed in XRD analysis. 

**Figure 3:**
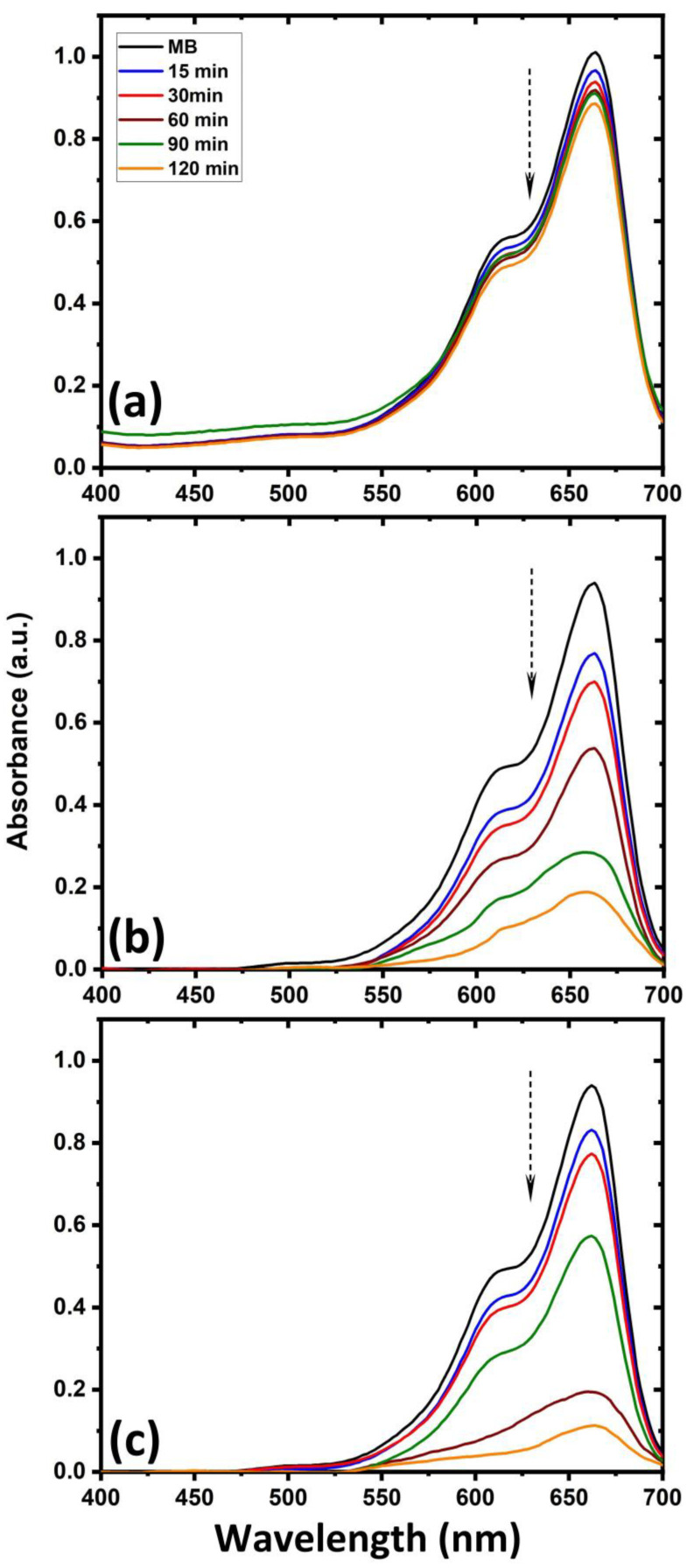
UV–visible spectrum for photodegradation of methylene blue over cotton fabric: (**a**) untreated material, (**b**) ZnO-1 sample, and (**c**) ZnO-2 sample.

**Figure 4:**
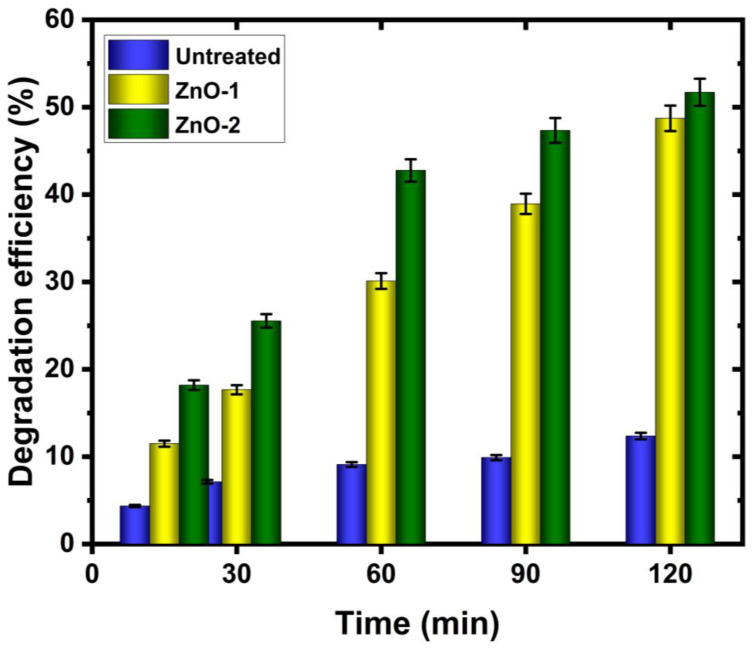
The photocatalytic degradation efficiency of methylene blue as a function of irradiation time in the presence of cotton fabric with and without ZnO deposition.

The photocatalytic degradation of methylene blue against ZnO-deposited cotton fabric after UV-light irradiation is also examined and the UV–visible spectrum is presented in Figure 3. The absorbance intensity of the methylene blue peak (664 nm) is not significantly changed for cotton fabric without ZnO deposition, whereas intensity decreases for ZnO-deposited samples when the irradiation time increases. The maximum degradation of methylene blue occurs for cotton fabric when ZnO is deposited using hydrogen–argon mixture discharge, which contains a higher amount of Zn (ZnO-2 sample). It suggests that ZnO-coated fabric, particularly when hydrogen gas is added to the argon plasma, is a suitable choice for the photocatalytic degradation of methylene blue [50,51]. The comparison of photocatalytic degradation efficiency of methylene blue as a function of irradiation time in the presence of cotton fabric with and without ZnO deposition is plotted in Figure 4. It shows that after 120 min of irradiation time, cotton fabric with ZnO deposition exhibits remarkable enhancement in photocatalytic degradation efficiency of methylene blue over cotton fabric without ZnO. It is evident that after 120 min, almost 88% of initial dyes still exist when the untreated cotton fabric is immersed in the solution. In contrast, only 48% of the initial dyes remained in the solution after the same immersion time when cotton fabric with ZnO (treated using hydrogen–argon mixture plasma) was immersed in the solution. 

**Figure 5:**
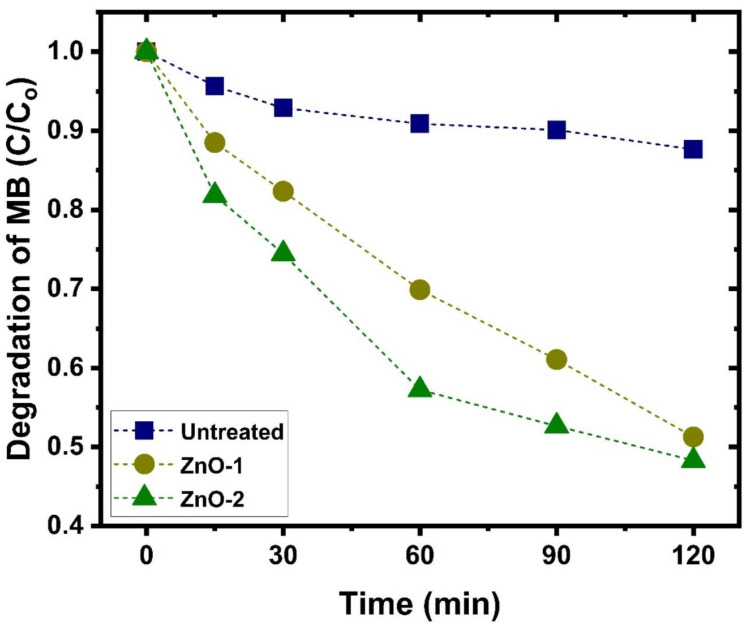
A plot of the degradation of methylene blue (C/Co) as a function of irradiation time in the presence of cotton fabric with and without ZnO deposition.

**Figure 6:**
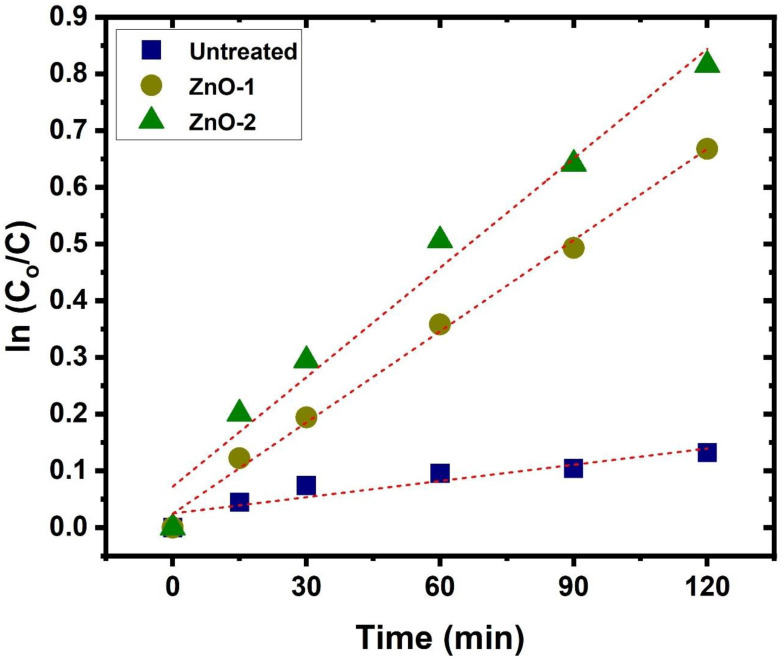
A plot of the photodegradation kinetics of methylene blue as a function of irradiation time in the presence of cotton fabric with and without ZnO deposition.

To understand the kinetic mechanism involved in the photocatalytic process of methylene blue, the graph among C/Co and irradiation time is plotted in Figure 5. It shows that the photodegradation proceeds gradually with the change in irradiation time, and a much steeper decrease is observed for cotton fabric with ZnO coating by hydrogen–argon gas plasma. It shows remarkable enhancement in the progradation of methylene blue by using ZnO-deposited cotton fabric, especially when hydrogen is added to the discharge. For better clarification of degradation rates of methylene blue against the cotton fabric with and without ZnO deposition, the rate constants are obtained from the well-known kinetic equation ln(C/Co) = kt, and the graph is depicted in Figure 6. The degradation rate constant is estimated by taking the linear fit of data points and the resultant slope of fit. The rate constants are 0.000954, 0.00536, and 0.00643 min^−1^ for untreated cotton fabric, ZnO deposition by argon gas, and hydrogen–argon gas plasma, which shows higher photocatalytic activity after ZnO deposition [52]. The graph shows a linear trend, demonstrating that methylene blue degradation obeys first-order kinetics, which follows the existing literature [53]. This trend could be ascribed to the higher surface area of ZnO nanoparticles deposited on the fabric due to their small size and the wide spectrum of sunlight used in the photocatalytic process. It enhances the number of active sites on the surface of the cotton fabric and thus causes a higher reactivity [52]. The mechanism involved in the improvement in the photocatalytic activity of cotton fabric by ZnO deposition is explained by Huang et al. [54], by which photogenerated (due to sunlight) electron–hole pairs are transferred to the surface of ZnO, which, as a result, react with oxygen O_2_ and H_2_O to produce O^2−^ and OH^−^ ions. These ions may play a role in the degradation of methylene blue through direct oxidation.

**Figure 7:**
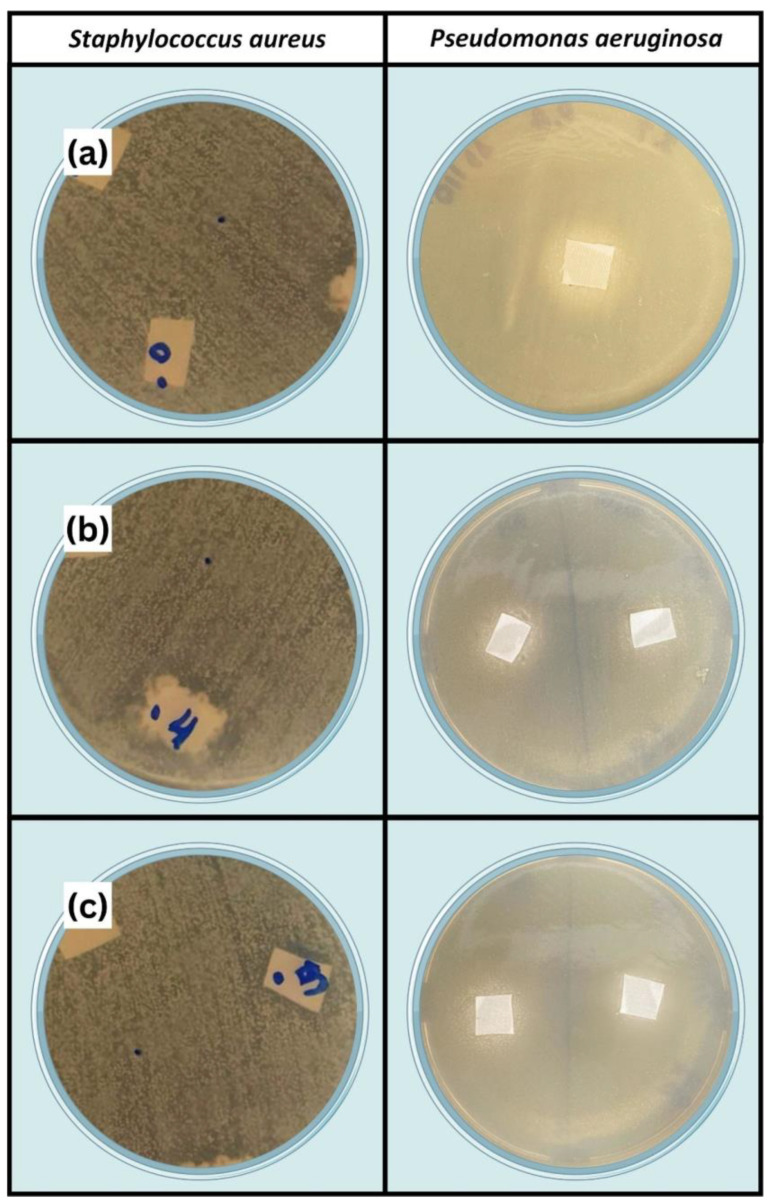
Antibacterial activity of cotton fabric with a zone of inhibition against different bacteria: (**a**) untreated fabric, (**b**) ZnO-1 sample, and (**c**) ZnO-2 sample.

**Figure 8:**
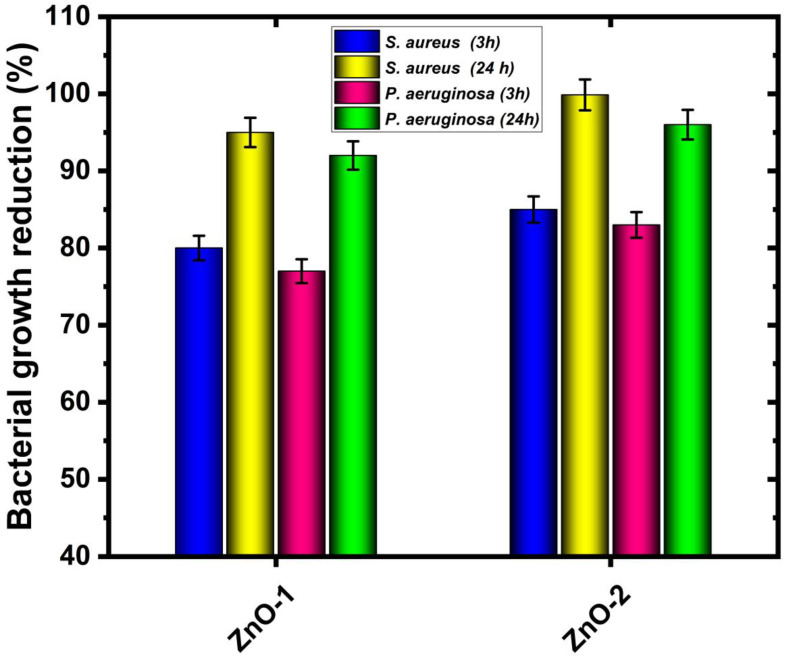
Bacterial growth reduction in the cotton fabric against different bacteria after ZnO deposition.

The antibacterial performance of ZnO-deposited cotton fabric for numerous medical uses is tested using the disk diffusion method. Figure 7 shows images of the zone of inhibition against different bacteria, *Staphylococcus aureus* (MRSA) and *Pseudomonas aeruginosa* (KPC+). It clearly shows that a zone of inhibition is induced when ZnO-deposited cotton fabric is placed on the disks, while the untreated cotton fabric does not show any inhibition zone. The diameter of the inhibition zone for ZnO-deposited cotton fabric using argon gas (ZnO-1) is 15.5 mm and 13.5 mm, and for hydrogen–argon gas (ZnO-2), it is 18.5 and 14.5 mm, for *Staphylococcus aureus* and *Pseudomonas aeruginosa* bacteria, respectively. In addition, the absorption method follows the ISO 20743:2010 standard, which is also used to examine the antibacterial performance of cotton fabric [55]. In this test, the cotton fabric is initially permitted to absorb bacterial inoculum, and later on, fabric and bacteria are incubated simultaneously, viable cells are enumerated after 24 h contact, and bacterial growth reduction is obtained, as presented in Figure 8. As observed in the images presented in Figure 7, no antibacterial effect is attained after 24 h of contact time for untreated cotton fabric. However, fabric with ZnO deposition shows a noteworthy antibacterial effect, and up to 99% reduction in bacterial growth of *Staphylococcus aureus* bacteria, and up to 97% for *Pseudomonas aeruginosa* (KPC+), is observed. Several authors explained the antibacterial performance of cotton fabric with ZnO deposition. They presented several possible justifications: Yamamoto et al. [55] specified that the formation of reactive oxygen species in the ZnO-deposited nanoparticles contributes to the bactericidal activity of ZnO. On the other hand, Zhang et al. [56] claimed that the antibacterial performance of ZnO nanoparticles is probably caused by the chemical interaction between membrane proteins and hydrogen peroxide. The hydrogen peroxide can enter the cell’s bacterial membrane and play a role in killing them. Another possibility is the chemical interaction among chemical species generated by the presence of ZnO and the outer lipid bilayer of bacteria. In addition, Padmavathy and Vijayaraghavan [57] justified that the antibacterial activity of ZnO is due to the formation of hydrogen peroxide, which is in contact with the killed bacteria and plays a role in stopping further bacterial growth. The obtained results in this study are well matched with the above reports and show that the deposition of ZnO on cotton fabric by CCPD can efficiently stop the bacterial growth of *Staphylococcus aureus* and *Pseudomonas aeruginosa bacteria,* which is of great clinical importance as a non-pharmacological method was used against two bacteria on the World Health Organization’s (WHO) list of priority pathogens. *Pseudomonas aeruginosa*, resistant to carbapenems, is in Priority 1 (critical), and *Staphylococcus aureus*, resistant to methicillin, is in Priority 2 (high); both bacteria were used in this research. 

## 3. Materials and Methods

### 3.1. Materials and Reagents

**Figure 9:**
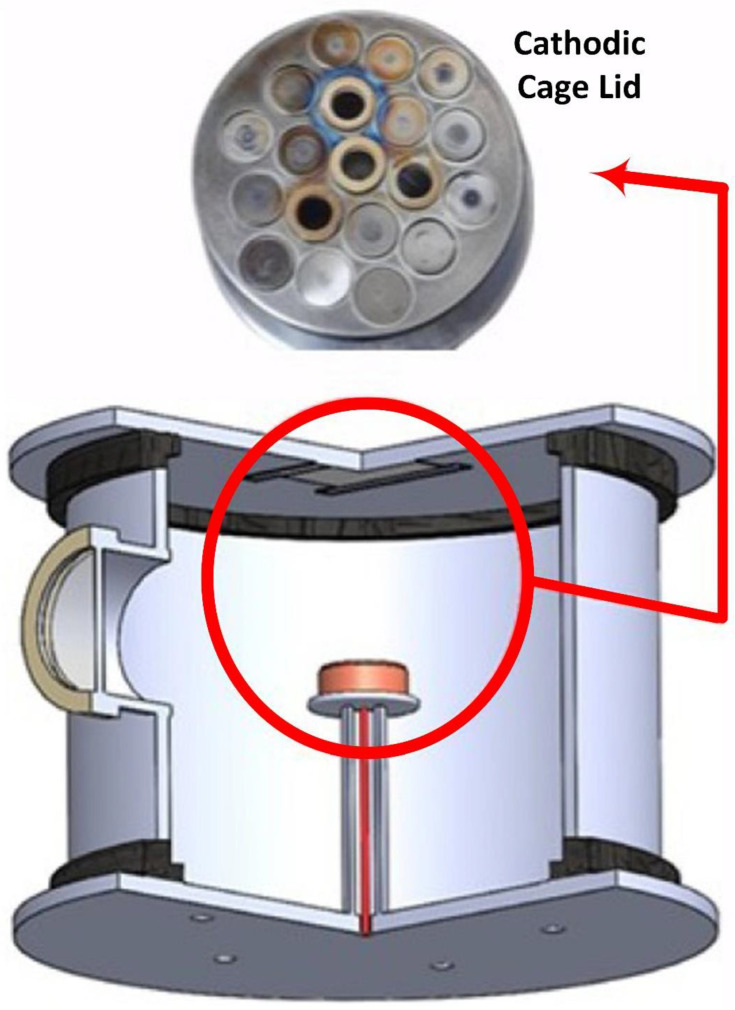
A schematic diagram of the cathodic cage plasma deposition system, where ZnO rings are inserted in the lid of the cathodic cage, and samples are placed on the lid of the chamber.

Here, cotton fabric (Sigma Aldrich, St. Louis, MO, USA) samples with a 2 × 1 twill structure, a weight of 338 g/m^2^, and a dimension of 13 × 7 cm^2^ were used to deposit ZnO. The deposition was carried out in a cathodic cage plasma deposition (CCPD) system, where samples were attached to the lid of the CCPD reactor instead of the sample holder to avoid overheating [34]. The cathodic cage was made of stainless steel, and for the deposition of ZnO, rings made of ZnO were inserted into the holes of the cathodic cage. The cage of stainless steel was 30 cm in height and 31 cm in diameter, and its lid consisted of uniformly distributed holes of 12 mm in diameter. The ZnO rings were prepared from 1.1 g of powdered zinc oxide (Sigma Aldrich), pressed with a hydraulic press with a load of 2 tons, and had a ring thickness of 1.9 mm and an external diameter of 11.8 mm for proper installation on the cage holes. Four compacted rings adjusted at the center of the lid with uniform distribution were used, and the remaining holes were covered with small disks made up of cage material, allowing plasma formation to occur mainly inside the zinc oxide rings for proper deposition, as clarified in Figure 9. Before treatment, the cathode cage was sanded under running water, dried, and cleaned with acetone to remove surface dirt. The internal sides of the reactor and the lid of the reactor were cleaned only with acetone. Then, the plasma was generated with a pressure of 0.5 mbar at a potential difference of 500 V, and the gas was injected into the reactor. The flow was fixed at 5 sccm for individual treatment with argon (ZnO-I) and hydrogen–argon (50:50 ratio) (ZnO-II) gas samples. Depending on the gas, the current was maintained at 0.09 and 0.10 A, and the treatment intervals were standardized at 1 h.

### 3.2. Characterization Techniques

The surface of the treated fabric was analyzed using scanning electron microscopy with a field emission electron beam (SEM-FEG) using the model Zeiss Auriga. This method also allows for the analysis of the chemical composition of the deposition through Energy Dispersive Spectroscopy (EDS). Grazing incidence X-ray diffraction (GIXRD) was also used to analyze the phase structure of the samples’ surfaces with the model Bruker D2 Phaser diffractometer. To analyze the photocatalytic properties of the samples, a dye solution was prepared, and the samples were inserted into a UV radiation exposure chamber. For this, methylene blue dye was used with a molecular weight equal to 373.02 g/mol solubilized in distilled water at a 2 × 10^−5^ mol/L concentration. Then, the samples were taken to a closed chamber with UV light for 2 h without shaking. To evaluate degradation as a function of time, after every 15 min, an amount of the solution was removed from each tissue, and the absorbance was analyzed using a spectrophotometer. Genesys 10 UV Scanning (Thermo Fisher Scientific, Waltham, MA, USA) from the Thermo Scientific brand was used for this test. The wavelength range analyzed was 400 to 700 nm.

The evaluation of bacterial growth and bactericidal properties in treated fabrics were qualitatively analyzed by the Mueller Hinton Agar Antibiogram using the Gram-positive bacteria methicillin-resistant *Staphylococcus aureus* (MRSA) and carbapenem-resistant *Pseudomonas aeruginosa* (KPC+). The cells were seeded on Mueller Hinton Agar, and the plates were incubated in a bacteriological oven at 37 °C to wait for bacterial development for 24 h. Several colonies were used to make the bacterial suspension in sterile 0.9% sodium chloride. The reading was adjusted to 0.73 ± 0.005 (equal to 1 McFarland), then some colonies were transferred to saline, homogenizing and reading 200 μL and an optical density of 600 nm in a 96-well plate. Bacteria concentration was assessed by optical density using a spectrophotometer. The bacteria were sown on nutrient agar using a swab in three directions in the Petri dish. Then, the treated tissues were placed and incubated in a bacteriological oven for 24 h at 37 °C. Crystal violet methodology was used to analyze antibacterial properties quantitatively. In a 24-well plate containing Mueller Hinton culture medium and methicillin-resistant *Staphylococcus aureus* (MRSA) and carbapenem-resistant *Pseudomonas aeruginosa* (KPC+), tissues were added in triplicate. Then, the treated tissue was placed and incubated in an oven for 24 h at 37 °C. Then, the medium was removed, and 1000 μL of 0.4% crystal violet was added for 15 min. An amount of 1000 μL of crystal violet was removed and discarded. Subsequently, washing was carried out in running water until no more dye emerged. An amount of 1000 μL of 99.5% dimethyl sulfoxide (DMSO) was added to all wells for 30 min. An amount of 200 μL was taken from each well into a 96-well plate.

## 4. Conclusions

In this work, a cathodic cage plasma deposition (CCPD) system is applied to deposit zinc oxide (ZnO) on cotton fabric to improve its photocatalytic and antibacterial performance. CCPD is commonly reported to be suitable for metallic substrates, but here, we proved that it is compatible with fabric samples, and overheating and the temperature sensitiveness of fabric can be prevented in CCPD. The main outcomes from this work are as follows:

1. Instead of conventional techniques, CCPD is an efficient choice for the deposition of ZnO on the cotton fabric, and it can be used for deposition in single-step processes, i.e., separate steps are not required for nanoparticle synthesis and subsequent deposition on fabric.
2. The XRD results showed that ZnO is deposited on fabric, which exhibits a hexagonal wurtzite structure.
3. The untreated cotton fabric shows that initial dyes still exist in the solution, but for ZnO-deposited cotton fabric, 52% of the initial dyes are degraded after the same immersion time. In particular, the ZnO deposition is efficient when a hydrogen–argon gas mixture is used in the CCPD system.
4. The antibacterial performance of fabric with ZnO deposition is remarkable, and a reduction in bacterial growth is observed (up to 99% for *Staphylococcus aureus* and 97% for *Pseudomonas aeruginosa* bacteria). In the antibacterial test, the ZnO deposition is again more efficient for the hydrogen–argon gas mixture in CCPD operation.

The successful deposition of ZnO by the CCPD technique provided an efficient and low-cost alternative. Specifically, in this system, no toxic chemicals are used to synthesize ZnO nanoparticles, multiple steps are not required for nanomaterial synthesis and deposition, and it is more advantageous. Last but not least, CCPD is already in use in industrial-scale applications, and thus, the outcomes of this study are expected to be of noteworthy importance for the garment sector and hospitals. 

## Data Availability

Data will be made available on request.
